# Risk of Second Primary Neoplasms Among Cancer Survivors: A Population‐Based, Cohort Study in Golestan Province, Northern Iran, 2004–2019

**DOI:** 10.1002/cam4.70926

**Published:** 2025-05-20

**Authors:** Susan Hasanpour‐Heidari, Shahryar Semnani, Abdolreza Fazel, Mohammad Naeimi‐Tabiei, Mahshid Mehrjerdian, SeyedMehdi Sedaghat, Hamideh Sadeghzadeh, Faezeh Salamat, Nastaran Jafari‐Delouei, Fatemeh Ghasemi‐Kebria, Honeyehsadat Mirkarimi, Nesa Shokouhifar, Behnoush Abedi‐Ardekani, Elisabete Weiderpass, Gholamreza Roshandel, Reza Malekzadeh

**Affiliations:** ^1^ Golestan Research Center of Gastroenterology and Hepatology Golestan University of Medical Sciences Gorgan Iran; ^2^ Cancer Research Center Golestan University of Medical Sciences Gorgan Iran; ^3^ Department of Pathology Golestan University of Medical Sciences Gorgan Iran; ^4^ Deputy of Public Health Golestan University of Medical Sciences Gorgan Iran; ^5^ International Agency for Research on Cancer (IARC) Lyon France; ^6^ Office of the Director International Agency for Research on Cancer (IARC) Lyon France; ^7^ Digestive Oncology Research Center, Digestive Diseases Research Institute Tehran University of Medical Sciences Tehran Iran

**Keywords:** cancer, Golestan, Iran, multiple primary neoplasm, second primary neoplasm

## Abstract

**Background:**

Recent reports of the Golestan population‐based cancer registry (GPCR) suggested increasing trends in the incidence and survival rates of cancers in Golestan, Northern Iran. We investigated the risk of developing second primary neoplasms (SPNs) among cancer survivors in Golestan.

**Methods:**

The GPCR cases for whom a first primary cancer was diagnosed between 2004 and 2019 were included as cohort participants. The cohort members were followed by the end of 2020, and the occurrence of a second primary neoplasm (SPN) was considered as the study outcome event. The standardized incidence ratios (SIRs) and the Absolute excess risks (AERs), with corresponding 95% confidence intervals (95% CI) were calculated to evaluate the risk of SPNs.

**Results:**

Of the total 32,980 cases with first primary cancer, with a median follow‐up of 3.4 years, 772 (2.3%) SPNs were registered. Our findings suggested a significantly higher risk of occurring new neoplasms among cancer survivors, with a SIR of 4.6 (95% CI: 4.3–4.9) and an AER of 41.8 per 10,000 person‐years (95% CI: 37.6–46.0). Rural residents had a higher risk of SPN (SIR = 5.48) than urban dwellers (SIR = 3.99). Patients with first primary cancers of the ovary (SIR = 6.83) and prostate (SIR = 6.72) had the highest risk of any SPNs. The highest risk of site‐specific SPNs was observed for the SPNs of the ovary (SIR = 8.11) and NHL (SIR = 7.07).

**Conclusions:**

Our results suggest that cancer patients are at significantly higher risk of getting a new neoplasm than the general population. These findings highlight the need for designing and implementing efficient surveillance programs for cancer survivors.

## 1 Introduction

Cancer is a significant global health challenge with rising incidence and prevalence. In 2020, there were 19.3 million new cancer cases and 9.9 million cancer‐related deaths reported worldwide [1]. These numbers are projected to increase even further in the coming years [2]. In addition, the 2019 Global burden of disease (GBD) study showed the growing burden of cancer in low and middle‐sociodemographic index (SDI) countries including Iran [3]. Iran is witnessing a growing trend in cancer incidence, and it is predicted that the number of cancer cases will have a 42.6% increase from 2016 to 2025 [4]. In the Golestan province, a high‐risk area for upper gastrointestinal cancers in Northern Iran [5, 6], this rate is even higher. The Golestan Population‐based Cancer Registry (GPCR) reported that the number of new cancer cases in 2025 is estimated to increase by 61.3% compared with 2016 statistics in this region [7].

These days, thanks to cancer prevention strategies, advances in diagnostic techniques, and high‐quality care for cancer patients, the trend of survival in cancer cases is increasing globally [8, 9]. Recent reports from the GPCR suggested an increase in survival rates of cancer patients among Golestan residents [10, 11]. Cancer survivors have a higher risk of multiple primary cancer (estimated 1.5 to 3.1 times higher) in comparison with the general population [12]. According to the International Agency for Research on Cancer (IARC) protocols, multiple primary cancers are defined as the occurrence of two or more distinct cancers in an individual, which is neither an extension, nor a recurrence, nor metastasis [13]. Statistics showed that the risk of multiple primary cancers is higher among patients with better survival chances, for example, breast cancer; this can be related to the cancer treatment late effects or common risk factors to the first primary cancers.

Therefore, it is expected that with an increase in the number of new cancer cases in the future (it is predicted to reach 30.2 million new cancer cases by 2040 globally) and the growing trend in survival rate generally, the risk of subsequent primary neoplasms is also likely to increase [2, 8, 14]. Therefore, investigating the risk of developing subsequent primary cancers is crucial for making effective plans for follow‐up in cancer patients [12, 15].

Considering recent reports of increasing trends in the incidence and survival rates of cancers in Golestan province, we conducted this study to investigate the risk of second primary neoplasms (SPNs) among cancer survivors in this high‐risk population, between 2004 and 2019.

## 2 Materials and Methods

### 2.1 Source of Data and Definitions

Data on cancer patients as well as data on SPN were obtained from the GPCR dataset. The GPCR started its activity as a high‐quality population‐based cancer registry in 2004 [7, 16]. It covers the population of Golestan province, with 1.87 million residents in 2016 [17], constituting 2.3% of Iran's total population, located in Northern Iran with an area of about 20,000 km^2^. The GPCR was accepted as a voting member of the International Association of Cancer Registries (IACR) in 2007, and its data were accepted for publication in volumes X, XI, and XII of the Cancer Incidence in Five Continents (CI‐5) project of the IARC [18, 19, 20]. Based on the indices of data quality, the GPCR is a high‐quality population‐based cancer registry which collects data on cancer patients with acceptable accuracy and completeness across sub‐populations of the Golestan province (e.g., urban and rural populations) [16, 21].

The GPCR registered subsequent primary neoplasms (multiple primary cancers) in cancer patients using the IARC “international rules for multiple primary cancers” [13, 15, 22]. According to the IARC rules, multiple primary cancers are defined and registered as occurring two or more primary neoplasms in a person that originate in the primary site or tissue and are not extension, recurrence, or metastasis. Based on the number of multiple primary cancers in the GPCR dataset, only SPN were included in the present study. In other words, we excluded third primary neoplasms due to the very small sample size in the GPCR dataset.

The GPCR considered a passive follow‐up for its registered cases by performing linkage between the GPCR dataset and the Golestan death registry dataset [16, 23]. We obtained follow‐up data, including vital status and date of death, from the GPCR dataset.

### 2.2 Study Cohort and Follow‐Up

The GPCR cases for whom a first primary cancer was diagnosed between 2004 and 2019 were included as our cohort participants. Patients with non‐malignant tumors were excluded. We also excluded cases with non‐melanoma skin cancer (NMSC). Based on the availability of follow‐up data in the GPCR dataset, our cohort members were followed until the end of 2020. Person‐years at risk were accumulated for each of our cohort participants from the date of diagnosis of the first primary cancer up to the earliest of three events: date of diagnosis of an SPN, date of death, or end of follow‐up (December 31, 2020).

### 2.3 Explanatory Variables

According to the availability of data, we stratified all analyses by sex, residence area, year of diagnosis of first cancer, age at diagnosis of first cancer, and cancer site. The year of diagnosis of the first cancer was categorized into 3 periods including 2004–2009, 2010–2014, and 2015–2019. Considering age at diagnosis of the first primary cancers, participants were categorized into children (0–14 years), adolescents and young adults (AYA) (15–39 years), and adults (≥ 40 years). The age categorization was made according to the previous studies and based on differences in tumor characteristics and behaviors between the selected age groups [12, 24, 25, 26]. For site‐specific analysis, cancer sites were classified based on the International Classification of Disease, tenth revision (ICD‐10). According to the most recent reports of the GPCR [7, 16, 27], the following 10 most common cancer sites, including breast (female) (C50), stomach (C16), esophagus (C15), colorectum (C18‐C20), lung (C34), leukemia (C91‐C95), brain (C70‐C72), prostate (C61), bladder (C67), non‐Hodgkin lymphoma (NHL) (C82‐C85, C96), thyroid (C73), liver (C22), ovary (C56), larynx (C32), and pancreas (C25) were considered for site‐specific analysis.

### 2.4 Statistical Analysis

#### 2.4.1 Incidence Rates of First Primary Cancer in the General Population

The numbers and incidence rates of first primary cancer in the general population of Golestan (2004–2019) were presented across sex, residence area, period of diagnosis, age groups, and cancer sites. Crude incidence rates in the general population (General incidence rates) were calculated by dividing the number of first primary cancer cases by the corresponding number of the general population of Golestan. Age standardized incidence rates (ASR) of first primary cancer were calculated by the direct standardization method using the World standard population [28].

#### 2.4.2 Assessing the Risk of SPN

The median and interquartile range (IQR) were calculated for overall follow‐up time and time to SPN. The observed numbers of SPNs among the study cohort were presented by sex, residence area, period of diagnosis, age group, and cancer site. To evaluate the risk of SPN, we calculated the standardized incidence ratios (SIRs) and the Absolute excess risks (AERs). The main aim of the calculation of SIRs is to measure the strength of association between first and second primary cancers. The AERs are calculated to measure the absolute increase in risk of a SPN.

As a requirement for the calculation of SIRs and AERs, we needed to calculate the expected number of SPNs among the study cohort. The expected number of SPNs was calculated by applying the general incidence rates (incidence rates of first primary cancers in general population of Golestan) to the person‐years at risk among the study cohort stratified by sex, age group, and cancer site.

SIRs were calculated as the ratio of the observed and the expected number of SPNs. The SIRs were stratified by sex, residence area, period of diagnosis, age group, and cancer site. We also calculated the 95% confidence intervals (95% CIs) for the SIRs assuming a Poisson distribution [29]. AERs were calculated as the observed minus the expected number of SPNs, divided by the person‐years at risk and multiplied by 10,000. The AERs were stratified by sex, residence area, period of diagnosis, age group, and cancer site. The 95% CIs for AERs were calculated according to the previously described method, assuming a normal distribution [30]. SIRs and AERs were considered to be statistically significant if the 95% CIs did not include 1 and 0, respectively. In the case of fewer than 5 observed SPNs, the SIR and AER were not calculated, because the results were not reliable due to the small number of SPNs. Statistical analyses were performed using R software (version 4.3.1).

### 2.5 Ethical Considerations

The protocol of this study was reviewed and approved by the ethics committee of the Golestan University of Medical Sciences (ethics code: IR.GOUMS.REC.1400.212).

## 3 Results

Our study cohort comprises 32,980 cases with first primary cancers (excluding NMSC) diagnosed between 2004 and 2019. The crude incidence rate (general incidence rate) and the ASR of first primary cancers in the Golestan population were 117.0 and 144.2 (95% CI: 142.5–145.8) per 100,000 person‐years. Of the total 32,980 first primary cancers, 17,030 (51.61%), 18,206 (55.2%), and 26,981 (81.8%) occurred in males, urban residents, and adults, respectively. The most common sites of first primary cancers were the breast (*n* = 4206, 12.8%), stomach (3699, 11.2%), and esophagus (3093, 8.4%). Table 1 gives more detailed information on the characteristics of our study cohort.

**TABLE 1:** Cohort characteristics: Number (*N*), percent (%), crude incidence rate (general rate) (per 100,000), and ASR (per 100,000 person‐years) of cancer patients (excluding nonmelanoma skin cancer) in Golestan, Iran, 2004–2019.

Variables	Both male and female				Male				Female			
	*N*	%	General rate	ASR (95% CI)	*N*	%	General rate	ASR (95% CI)	*N*	%	General rate	ASR (95% CI)
Total	32,980	100.00	116.96	144.15 (142.54–145.76)	17,030	100.00	120.80	156.85 (154.42–159.28)	15,950	100.00	113.11	131.97 (129.85–134.09)
Age group												
Children	775	2.35	10.32	10.36 (9.63–11.09)	463	2.72	12.10	12.16 (11.04–13.28)	312	1.96	8.47	8.50 (7.56–9.44)
AYA	5224	15.84	39.69	38.17 (37.13–39.21)	2004	11.77	30.59	29.73 (28.42–31.04)	3220	20.19	48.70	46.50 (44.89–48.11)
Adults	26,981	81.81	358.52	396.29 (391.47–401.11)	14,563	85.51	391.51	444.01 (436.62–451.40)	12,418	77.86	326.27	350.42 (344.15–356.69)
Residence area												
Urban	18,206	55.20	125.38	150.35 (148.08–152.62)	9045	53.11	124.50	158.44 (155.05–161.83)	9161	57.44	126.25	142.92 (139.88–145.96)
Rural	14,774	44.80	108.02	137.46 (135.17–139.75)	7985	46.89	116.87	156.48 (152.91–160.05)	6789	42.56	99.19	119.54 (116.62–122.46)
Calendar period												
2004–2009	10,045	30.46	102.51	140.05 (137.19–142.91)	5352	31.43	109.79	154.08 (149.75–158.41)	4693	29.42	95.31	125.45 (121.69–129.21)
2010–2014	9851	29.87	109.91	134.49 (131.73–137.25)	5207	30.58	116.24	151.02 (146.75–155.29)	4644	29.12	103.57	119.11 (115.56–122.66)
2015–2019	13,084	39.67	138.66	155.88 (153.14–158.62)	6471	38.00	136.42	164.42 (160.28–168.56)	6613	41.46	140.92	148.91 (145.23–152.59)
Site of first cancer												
Esophagus	3093	9.38	10.97	14.90 (14.37–15.43)	1742	10.23	12.36	16.97 (16.15–17.79)	1351	8.47	9.58	12.91 (12.20–13.62)
Stomach	3699	11.22	13.12	17.54 (16.95–18.13)	2549	14.97	18.08	24.73 (23.73–25.73)	1150	7.21	8.16	10.59 (9.96–11.22)
Colorectum	3056	9.27	10.84	13.62 (13.13–14.11)	1693	9.94	12.01	15.49 (14.73–16.25)	1363	8.55	9.67	11.83 (11.18–12.48)
Liver	739	2.24	2.62	3.45 (3.20–3.70)	476	2.80	3.38	4.55 (4.12–4.98)	263	1.65	1.87	2.40 (2.11–2.69)
Pancreas	580	1.76	2.06	2.76 (2.52–3.00)	362	2.13	2.57	3.53 (3.16–3.90)	218	1.37	1.55	2.03 (1.76–2.30)
Larynx	590	1.79	2.09	2.73 (2.49–2.97)	460	2.70	3.26	4.36 (3.95–4.77)	130	0.82	0.92	1.17 (0.95–1.39)
Lung	2403	7.29	8.52	11.29 (10.82–11.76)	1675	9.84	11.88	16.20 (15.40–17.00)	728	4.56	5.16	6.61 (6.12–7.10)
Leukemia	1963	5.95	6.96	8.07 (7.70–8.44)	1144	6.72	8.12	9.54 (8.97–10.11)	819	5.13	5.81	6.63 (6.16–7.10)
Breast	4206	12.75	14.92	16.00 (15.49–16.51)	88	0.52	0.62	0.75 (0.59–0.91)	4118	25.82	29.20	30.88 (29.90–31.86)
Ovary	727	2.20	2.58	2.93 (2.71–3.15)	0	0.00	0.00	0.00 (0.00–0.00)	727	4.56	5.16	5.77 (5.34–6.20)
Prostate	1316	3.99	4.67	6.45 (6.10–6.80)	1316	7.73	9.34	13.03 (12.30–13.76)	0	0.00	0.00	0.00 (0.00–0.00)
Bladder	1241	3.76	4.40	5.81 (5.48–6.14)	977	5.74	6.93	9.34 (8.73–9.95)	264	1.66	1.87	2.39 (2.10–2.68)
Brain	1434	4.35	5.09	5.76 (5.45–6.07)	813	4.77	5.77	6.58 (6.11–7.05)	621	3.89	4.40	4.97 (4.56–5.38)
Thyroid	801	2.43	2.84	2.89 (2.67–3.11)	186	1.09	1.32	1.50 (1.26–1.74)	615	3.86	4.36	4.25 (3.90–4.60)
NHL	1128	3.42	4.00	4.68 (4.39–4.97)	705	4.14	5.00	5.96 (5.51–6.41)	423	2.65	3.00	3.43 (3.10–3.76)
Other	6004	18.20	21.29	25.25 (24.58–25.92)	2844	16.70	20.17	24.32 (23.38–25.26)	3160	19.81	22.41	26.13 (25.19–27.07)

Abbreviations: 95% CI, 95% confidence interval; ASR, age‐standardized incidence rate.

Overall, the study cohort contributed 144,321.8 person‐years of follow‐up between January 1, 2004, and December 31, 2020 (median follow‐up 3.4 years; IQR: 0.9–8.0). Table 2 shows the person‐years of follow‐up across age groups, sex, residence area, period of diagnosis, and cancer sites.

**TABLE 2:** Numbers (observed and expected) and risks (SIR and AER) of second primary neoplasms (SPNs) in a cohort of cancer patients (excluding non‐melanoma skin cancer) in Golestan, Iran, 2004–2019, by age group, place of residence, period of diagnosis, and site of cancer.

Variables	Both male and female				Male				Female			
	PY	Observed/expected	SIR (95% CI)	AER (95% CI)	PY	Observed/expected	SIR (95% CI)	AER (95% CI)	PY	Observed/expected	SIR (95% CI)	AER (95% CI)
Total	144,321.8	772/168.8	**4.57** (4.25–4.90)	**41.80** (37.63–45.96)	69,544.4	378/84.0	**4.50** (4.05–4.95)	**42.28** (36.22–48.33)	74,777.4	394/84.6	**4.66** (4.20–5.12)	**41.38** (35.64–47.11)
Age group												
Children	4314.7	9/0.4	**22.50** (7.80–37.20)	**19.93** (6.00–33.86)	2617.6	7/0.3	**23.33** (6.05–40.62)	**25.60** (5.37–45.83)	1697.1	2/0.1	—^a^	—^a^
AYA	27,810.5	120/11.0	**10.91** (8.96–12.86)	**39.19** (31.13–47.26)	10,158.8	47/3.1	**15.16** (10.83–19.50)	**43.21** (29.56–56.87)	17,651.7	73/8.6	**8.49** (6.54–10.44)	**36.48** (26.45–46.51)
Adults	112,196.6	643/402.2	**1.60** (1.48–1.72)	**21.46** (15.81–27.11)	56,768.0	324/222.3	**1.46** (1.30–1.62)	**17.92** (9.85–25.98)	55,428.6	319/180.8	**1.76** (1.57–1.96)	**24.93** (17.03–32.84)
Residence area												
Urban	89,396.6	447/112.1	**3.99** (3.62–4.36)	**37.46** (32.28–42.65)	42,360.7	206/52.7	**3.91** (3.38–4.44)	**36.19** (28.75–43.63)	47,036.0	241/59.4	**4.06** (3.55–4.57)	**38.61** (31.39–45.83)
Rural	54,925.2	325/59.3	**5.48** (4.88–6.08)	**48.37** (41.38–55.37)	27,183.7	172/31.8	**5.41** (4.60–6.22)	**51.57** (41.28–61.87)	27,741.5	153/27.5	**5.56** (4.68–6.45)	**45.24** (35.75–54.73)
Calendar period												
2004–2009	76,346.4	251/78.3	**3.21** (2.81–3.60)	**22.62** (17.96–27.28)	37,169.0	124/40.8	**3.04** (2.50–3.57)	**22.38** (15.61–29.15)	39,177.3	127/37.3	**3.40** (2.81–4.00)	**22.90** (16.48–29.31)
2010–2014	42,615.6	225/46.8	**4.81** (4.18–5.44)	**41.82** (34.23–49.40)	20,664.2	112/24.0	**4.67** (3.80–5.53)	**42.59** (31.52–53.65)	21,951.3	113/22.7	**4.98** (4.06–5.90)	**41.14** (30.74–51.54)
2015–2019	25,359.9	296/35.2	**8.41** (7.45–9.37)	**102.8**4 (88.77–116.91)	11,711.2	142/16.0	**8.88** (7.42–10.33)	**107.59** (86.55–128.63)	13,648.7	154/19.2	**8.02** (6.75–9.29)	**98.76** (79.86–117.66)
Risk of any SPNs, by site of first cancer												
Esophagus	12,495.0	45/14.6	**3.08** (2.18–3.98)	**24.33** (12.22–36.44)	6693.0	29/8.1	**3.5**8 (2.28–4.88)	**31.23** (13.39–49.06)	5802.0	16/6.6	**2.42** (1.24–3.61)	**16.20** (0.14–32.26)
Stomach	13,134.4	54/15.4	**3.51** (2.57–4.44)	**29.39** (16.96–41.82)	8800.6	38/10.6	**3.58** (2.45–4.72)	**31.13** (15.61–46.66)	4333.8	16/4.9	**3.27** (1.67–4.87)	**25.61** (4.94–46.29)
Colorectum	13,768.4	85/16.1	**5.28** (4.16–6.40)	**50.04** (35.73–64.36)	7478.3	38/9.0	**4.22** (2.88–5.56)	**38.78** (20.81–56.75)	6290.1	47/7.1	**6.62** (4.73–8.51)	**63.43** (40.51–86.35)
Liver	2021.3	14/2.4	**5.83** (2.78–8.89)	**57.39** (18.12–96.66)	1197.0	9/1.4	**6.43** (2.23–10.63)	**63.49** (10.69–116.29)	824.3	5/0.9	5.56 (0.69–10.43)	49.74 (−8.02–107.50)
Pancreas	1505.1	12/1.8	**6.67** (2.89–10.44)	**67.77** (19.39–116.15)	904.2	8/1.1	**7.27** (2.23–12.31)	**76.31** (10.92–141.69)	600.9	4/0.7	—^a^	—^a^
Larynx	2555.5	18/3.0	**6.00** (3.23–8.77)	**58.70** (23.55–93.84)	2051.2	16/2.5	**6.40** (3.26–9.54)	**65.82** (24.72–106.91)	504.3	2/0.6	—^a^	—^a^
Lung	8851.5	48/10.4	**4.62** (3.31–5.92)	**42.48** (25.56–59.40)	5869.8	31/7.1	**4.37** (2.83–5.90)	**40.72** (20.11–61.33)	2981.7	17/3.4	**5.00** (2.62–7.38)	**45.61** (15.92–75.30)
Leukemia	8233.7	32/9.6	**3.33** (2.18–4.49)	**27.21** (11.85–42.56)	4782.2	20/5.8	**3.45** (1.94–4.96)	**29.69** (8.88–50.51)	3451.6	12/3.9	**3.08** (1.34–4.82)	**23.47** (0.82–46.11)
Breast	21,936.3	89/25.7	**3.46** (2.74–4.18)	**28.86** (19.29–38.43)	408.8	4/0.5	—	—	21,527.4	85/24.3	**3.50** (2.75–4.24)	**28.20** (18.68–37.72)
Ovary	3494.0	28/4.1	**6.83** (4.30–9.36)	**68.40** (36.62–100.19)	—	—	—	—	3494.0	28/4.0	**7.00** (4.41–9.59)	**68.69** (36.96–100.42)
Prostate	5508.2	43/6.4	**6.72** (4.71–8.73)	**66.45** (41.44–91.46)	5508.2	43/6.7	**6.42** (4.50–8.34)	**65.90** (40.82–90.99)	—	—	—	—
Bladder	6374.0	41/7.5	**5.4**7 (3.79–7.14)	**52.56** (31.14–73.97)	4934.7	31/6.0	**5.17** (3.35–6.99)	**50.66** (26.50–74.82)	1439.3	10/1.6	**6.25** (2.38–10.12)	**58.36** (11.98–104.74)
Brain	6343.4	23/7.4	**3.11** (1.84–4.38)	**24.59** (7.56–41.63)	3586.4	9/4.3	2.09 (0.73–3.46)	13.11 (−6.83–33.04)	2757.0	14/3.1	**4.52** (2.15–6.88)	**39.54** (10.14–68.93)
Thyroid	3996.9	12/4.7	**2.55** (1.11–4.00)	18.26 (−1.78–38.30)	868.3	6/1.0	**6.0**0 (1.20–10.80)	57.58 (−2.14–117.31)	3128.6	6/3.5	1.71 (0.34–3.09)	7.99 (−11.32–27.30)
NHL	5639.9	37/6.6	**5.61** (3.80–7.41)	**53.90** (30.95–76.85)	3495.7	22/4.2	**5.24** (3.05–7.43)	**50.92** (22.22–79.62)	2144.2	15/2.4	**6.25** (3.09–9.41)	58.76 (20.63–96.89)
Other	28,464.2	191/33.3	**5.74** (4.92–6.55)	**55.40** (45.09–65.72)	12,965.9	74/15.7	**4.71** (3.64–5.79)	**44.96** (30.65–59.28)	15,498.2	117/17.5	**6.69** (5.47–7.90)	**64.20** (49.53–78.87)
Risk of site‐specific SNPs in cancer survivors (i.e., by site of second neoplasm)												
Esophagus	144,321.8	53/15.8	**3.35** (2.45–4.26)	**2.58** (1.45–3.70)	69,544.4	30/8.6	**3.49** (2.24–4.74)	**3.08** (1.33–4.83)	74,777.4	23/7.2	**3.19** (1.89–4.50)	**2.11** (0.67–3.55)
Stomach	144,321.8	89/18.9	**4.71** (3.73–5.69)	**4.86** (3.45–6.27)	69,544.4	48/12.6	**3.81** (2.73–4.89)	**5.09** (2.90–7.28)	74,777.4	41/6.1	**6.72** (4.66–8.78)	**4.67** (2.87–6.47)
Colorectum	144,321.8	79/15.6	**5.06** (3.95–6.18)	**4.39** (3.07–5.71)	69,544.4	37/8.4	**4.40** (2.99–5.82)	**4.11** (2.21–6.01)	74,777.4	42/7.2	**5.83** (4.07–7.60)	**4.65** (2.82–6.49)
Liver	144,321.8	13/3.8	**3.42** (1.56–5.28)	**0.64** (0.08–1.19)	69,544.4	5/2.4	2.08 (0.26–3.91)	0.37 (−0.39–1.14)	74,777.4	8/1.4	**5.71** (1.75–9.67)	0.**88** (0.08–1.69)
Pancreas	144,321.8	12/3.0	**4.00** (1.74–6.26)	**0.62** (0.10–1.15)	69,544.4	7/1.8	**3.89** (1.01–6.77)	0.75 (−0.09–1.58)	74,777.4	5/1.2	4.17 (0.51–7.82)	0.51 (−0.14–1.16)
Larynx	144,321.8	15/3.0	**5.00** (2.47–7.53)	0.**83** (0.26–1.41)	69,544.4	12/2.3	**5.22** (2.27–8.17)	**1.39** (0.33–2.46)	74,777.4	3/0.7	—^a^	—^a^
Lung	144,321.8	37/12.3	**3.01** (2.04–3.98)	**1.71** (0.76–2.67)	69,544.4	26/8.3	**3.13** (1.93–4.34)	**2.55** (0.89–4.20)	74,777.4	11/3.9	**2.82** (1.15–4.49)	0.95 (−0.06–1.96)
Leukemia	144,321.8	42/10.0	**4.20** (2.93–5.47)	**2.22** (1.24–3.20)	69,544.4	26/5.6	**4.64** (2.86–6.43)	**2.93** (1.35–4.52)	74,777.4	16/4.3	**3.72** (1.90–5.54)	**1.56** (0.38–2.75)
Breast	144,321.8	79/21.5	**3.67** (2.86–4.48)	**3.98** (2.62–5.35)	69,544.4	7/0.4	**17.50** (4.54–30.46)	0.**95** (0.18–1.72)	74,777.4	72/21.8	**3.30** (2.54–4.07)	**6.71** (4.17–9.25)
Ovary	144,321.8	30/3.7	**8.11** (5.21–11.01)	**1.82** (1.03–2.61)	—	—	—	—	74,777.4	30/3.9	**7.69** (4.94–10.44)	**3.49** (1.96–5.02)
Prostate	144,321.8	36/6.7	**5.37** (3.62–7.13)	**2.03** (1.14–2.92)	69,544.4	36/6.5	**5.54** (3.73–7.35)	**4.24** (2.40–6.08)	—	—	—	—
Bladder	144,321.8	33/6.4	**5.16** (3.40–6.92)	**1.84** (0.99–2.70)	69,544.4	17/4.8	**3.54** (1.86–5.23)	**1.75** (0.44–3.07)	74,777.4	16/1.4	**11.43** (5.83–17.03)	**1.95** (0.86–3.05)
Brain	144,321.8	19/7.3	**2.60** (1.43–3.77)	0.**81** (0.11–1.51)	69,544.4	8/4.0	2.00 (0.61–3.39)	0.58 (−0.40–1.55)	74,777.4	11/3.3	**3.33** (1.36–5.30)	**1.03** (0.04–2.02)
Thyroid	144,321.8	13/4.1	**3.17** (1.45–4.89)	0.**62** (0.06–1.18)	69,544.4	3/0.9	—^a^	—^a^	74,777.4	10/3.3	**3.03** (1.15–4.91)	0.90 (−0.06–1.85)
NHL	144,321.8	41/5.8	**7.07** (4.91–9.23)	**2.44** (1.51–3.37)	69,544.4	24/3.5	**6.86** (4.11–9.60)	**2.95** (1.47–4.43)	74,777.4	17/2.2	**7.73** (4.05–11.40)	**1.98** (0.83–3.13)
Other	144,321.8	181/30.7	**5.90** (5.04–6.75)	**10.41** (8.44–12.39)	69,544.4	92/14.0	**6.57** (5.23–7.91)	**11.22** (8.31–14.12)	74,777.4	89/16.8	**5.30** (4.20–6.40)	**9.66** (6.96–12.35)

*Note:* Significant SIRs and AERs are marked in bold.

Abbreviations: 95% CI: 95% confidence interval; AER, absolute excess risks; AYA, adolescent and young adult; NHL, non‐Hodgkin lymphoma; PY, person‐years at risk; SIR, standardized incidence ratio.

^a^ SIR and AER were not calculated if the observed number of SNPs was < 5.

Of the 32,980 participants of the cohort, 772 (2.3%) experienced a SPN with the median time to SPN of 1.5 years (IQR: 0.4–4.2). The observed and expected number of SPNs, the SIRs, and the AERs across sex, residence area, period of diagnosis, age groups, and cancer sites are presented in Table 2. Our results showed a significantly higher risk of occurring new primary neoplasm in cancer patients when compared to the general population. The overall relative risk of developing a new primary cancer was 4.57 times higher among our cancer patients compared with the general population of Golestan (SIR = 4.57; 95% CI: 4.25–4.90). Our findings suggested that our cohort participants experienced 42 more (excess) primary neoplasms (per 10,000 person‐years of follow up) than the general population (AER = 41.80 per 10,000; 95% CI: 37.63–45.96).

Of the total 772 SPNs, 378 (49.0%), 447 (57.9%), and 643 (83.3%) were diagnosed in men, rural residents, and adults, respectively. The most common sites of SPNs were stomach (*n* = 89, 11.5%), colorectum (79, 10.2%), breast (79, 10.2%), and esophagus (53, 6.9%). The SIRs and AERs were similar for males (SIR = 4.50, AER = 42.28) and females (4.66, 41.38). Our findings suggested higher SIRs and AERs for rural residents (SIR = 5.48, AER = 48.37) than for urban residents (3.99, 37.46). The SIRs (95% CIs) for children, AYA, and adult population were 22.5 (95% CIs: 7.80–37.20), 10.91 (8.96–12.86), and 1.60 (1.48–1.72), and the corresponding AERs (95% CIs) were 19.93 (6.00–33.86), 39.19 (31.13–47.26), and 21.46 (15.81–27.11), respectively.

When assessing the risk of any SPN by the site of the first primary cancer, the highest SIRs of any SPN were found for patients with first primary cancer of ovary (SIR = 6.83; 95% CI: 4.30–9.36) and prostate (SIR = 6.72; 95% CI: 4.71–8.73). When assessing the risk of site‐specific SPN (i.e., by the site of the second primary neoplasm) among the whole cohort, the highest SIRs were observed for the SPNs of ovary (SIR = 8.11; 95% CI: 5.21–11.01) and NHL (SIR = 7.07; 4.91–9.23) (Table 2).

Table 3 shows the risk of SPNs by site of first primary cancer and site of SPN among AYA and adult population. Tables S1 and S2 show similar findings by gender for AYA and adult population, respectively. The highest SIRs of any SPN were found for patients with first primary cancer of lung (SIR = 16.67; 95% CI: 2.06–31.28) and ovary (SIR = 2.61; 95% CI: 1.55–3.68) among AYA and adult cancer patients, respectively. When assessing the risk of site‐specific SPN (i.e., by the site of the second primary neoplasm) in AYA and adult population, the highest SIRs were observed for the SPNs of NHL (SIR = 23.33; 95% CI: 11.11–35.56) and ovary (SIR = 3.09; 95% CI: 1.88–4.30), respectively.

**TABLE 3:** Numbers (observed and expected) and risks (SIR and AER) of second primary neoplasms (SPNs) in a cohort of cancer patients (excluding nonmelanoma skin cancer) in Golestan, Iran, 2004–2019, by sites of first and second cancer and age group.

Variables	Adolescent and young adults (AYA)				Adults			
	PY	Observed/expected	SIR (95% CI)	AER (95% CI)	PY	Observed/expected	SIR (95% CI)	AER (95% CI)
Risk of any SPNs by site of first cancer								
Esophagus	482.7	2/0.2	—^a^	—^a^	12,012.3	43/43.1	1.00 (0.70–1.30)	−0.08 (−15.22–15.06)
Stomach	680.0	4/0.3	—^a^	—^a^	12,454.4	50/44.7	1.12 (0.81–1.43)	4.26 (−11.06–19.57)
Colorectum	2165.9	11/0.9	**12.22** (5.00–19.44)	**46.63** (15.42–77.85)	11,600.6	74/41.6	**1.78** (1.37–2.18)	**27.93** (9.76–46.10)
Liver	170.1	1/0.1	—^a^	—^a^	1778.6	13/6.4	2.03 (0.93–3.14)	37.11 (−11.43–85.65)
Pancreas	69.6	1/0.0	—^a^	—^a^	1435.5	11/5.1	2.16 (0.88–3.43)	41.10 (−13.69–95.89)
Larynx	180.1	1/0.1	—^a^	—^a^	2367.3	17/8.5	**2.00** (1.05–2.95)	35.91 (−5.90–77.71)
Lung	808.8	5/0.3	**16.67** (2.06–31.28)	**58.11** (2.32–113.89)	8042.7	43/28.8	**1.49** (1.05–1.94)	17.66 (−2.99–38.31)
Leukemia	1968.7	6/0.8	**7.50** (1.50–13.50)	**26.41** (0.45–52.38)	4376.8	24/15.7	1.53 (0.92–2.14)	18.96 (−9.25–47.18)
Breast	6121.1	20/2.4	**8.33** (4.68–11.99)	**28.75** (13.60–43.91)	15,813.1	69/56.7	1.22 (0.93–1.50)	7.78 (−6.12–21.67)
Ovary	994.3	5/0.4	**12.50** (1.54–23.46)	**46.27** (0.46–92.07)	2451.6	23/8.8	**2.61** (1.55–3.68)	**57.92** (12.84–103.00)
Prostate	0.0	0/0.0	—^a^	—^a^	5492.4	43/19.7	**2.18** (1.53–2.84)	**42.42** (14.17–70.68)
Bladder	562.6	2/0.2	—^a^	—^a^	5803.5	39/20.8	**1.88** (1.29–2.46)	**31.36** (5.24–57.48)
Brain	2156.0	6/0.9	**6.67** (1.33–12.00)	23.66 (−0.22–47.54)	3589.4	17/12.9	1.32 (0.69–1.94)	11.42 (−18.44–41.28)
Thyroid	1953.8	3/0.8	—^a^	—^a^	2007.1	9/7.2	1.25 (0.43–2.07)	8.97 (−30.34–48.27)
NHL	1618.1	8/0.6	**13.33** (4.09–22.57)	**45.73** (10.21–81.25)	3711.6	29/13.3	**2.18** (1.39–2.97)	**42.30** (7.95–76.64)
Other	7869.4	45/3.1	**14.52** (10.27–18.76)	**53.24** (35.97–70.52)	19,259.5	139/69.0	**2.01** (1.68–2.35)	**36.35** (21.67–51.02)
Risk of site‐specific SNPs in cancer survivors (i.e., by site of second neoplasm)								
Esophagus	27,810.5	3/0.2	—^a^	—^a^	112,196.6	50/44.6	1.12 (0.81–1.43)	0.48 (−1.22–2.18)
Stomach	27,810.5	9/0.4	**22.50** (7.80–37.20)	**3.09** (0.93–5.25)	112,196.6	80/52.2	**1.53** (1.20–1.87)	**2.48** (0.47–4.49)
Colorectum	27,810.5	9/0.8	**11.25** (3.90–18.60)	**2.95** (0.74–5.15)	112,196.6	70/39.8	**1.76** (1.35–2.17)	**2.69** (0.86–4.52)
Liver	27,810.5	4/0.1	—^a^	—^a^	112,196.6	9/9.9	0.91 (0.32–1.50)	−0.08 (−0.84–0.68)
Pancreas	27,810.5	1/0.1	—^a^	—^a^	112,196.6	11/8.3	1.33 (0.54–2.11)	0.24 (−0.53–1.01)
Larynx	27,810.5	3/0.1	—^a^	—^a^	112,196.6	12/8.2	1.46 (0.64–2.29)	0.34 (−0.45–1.12)
Lung	27,810.5	3/0.3	—^a^	—^a^	112,196.6	34/33.5	1.01 (0.67–1.36)	0.04 (−1.39–1.48)
Leukemia	27,810.5	10/1.0	**10.00** (3.80–16.20)	**3.24** (0.90–5.57)	112,196.6	29/17.4	**1.67** (1.06–2.27)	1.03 (−0.16–2.22)
Breast	27,810.5	18/2.4	**7.50** (4.04–10.96)	**5.61** (2.43–8.79)	112,196.6	61/46.1	1.32 (0.99–1.66)	1.33 (−0.48–3.14)
Ovary	27,810.5	5/0.4	**12.50** (1.54–23.46)	**1.65** (0.02–3.29)	112,196.6	25/8.1	**3.09** (1.88–4.30)	**1.51** (0.50–2.51)
Prostate	0.0	0/0.0	—^a^	—^a^	112,196.6	36/19.5	**1.85** (1.24–2.45)	**1.47** (0.17–2.77)
Bladder	27,810.5	2/0.2	—^a^	—^a^	112,196.6	31/17.2	**1.80** (1.17–2.44)	**1.23** (0.02–2.44)
Brain	27,810.5	6/0.9	**6.67** (1.33–12.00)	1.83 (−0.02–3.69)	112,196.6	13/13.3	0.98 (0.45–1.51)	−0.03 (−0.92–0.87)
Thyroid	27,810.5	5/0.8	6.25 (0.77–11.73)	1.51 (−0.19–3.21)	112,196.6	8/6.3	1.27 (0.39–2.15)	0.15 (−0.51–0.81)
NHL	27,810.5	14/0.6	**23.33** (11.11–35.56)	**4.82** (2.13–7.51)	112,196.6	26/11.9	**2.18** (1.35–3.02)	**1.26** (0.18–2.33)
Other	27,810.5	28/2.8	**10.00** (6.30–13.70)	**9.06** (5.15–12.97)	112,196.6	148/66.0	**2.24** (1.88–2.60)	**7.31** (4.75–9.86)

*Note:* Significant SIRs and AERs are marked in bold.

Abbreviations: 95% CI, 95% confidence interval; AER, absolute excess risks; NHL, non‐Hodgkin lymphoma; PY, person‐years at risk; SIR, standardized incidence ratio.

^a^ SIR and AER were not calculated if the observed number of SNPs was < 5.

## 4 Discussion

Our findings demonstrated a significantly elevated risk of developing a new neoplasm among cancer patients compared to the general population with an SIR of 4.5 and an AER of 41.8 per 10,000 person‐years. This higher risk of new primary neoplasm was similarly indicated in different studies [12, 14, 25, 31]. In a Canadian study, the SIR and AER among the children and youth (0–19 years) were calculated as 6.5 and 16.5 per 10,000 person‐years, respectively [26]. There are several explanations for the greater risk of new primary neoplasm among cancer survivors, including shared genetic susceptibilities, shared environmental factors, side effects of cancer therapies (e.g., radiotherapy, chemotherapy), and increased survival rate and life expectancy in cancer patients [15, 24].

Different types of cancers may have similar genetic susceptibilities. A 2023 study in the US reported that breast cancer patients carrying three genes, BRCA1, BRCA2, and ERCC2, were up to 56% more likely to be diagnosed with SPNs [32]. Genetic variants inherited can influence DNA damage repair mechanisms associated with diverse cancer predisposition syndromes, raising the risk of SPNs over time [33]. In addition to genetic factors, many environmental factors (e.g., smoking) are common risk factors for different types of cancers. Continued long‐term exposure to these risk factors in cancer survivors may result in a higher risk of subsequent primary neoplasms [34]. Although cancer therapies can improve prognosis in cancer patients, they may also lead to adverse effects, such as the development of SPNs [35]. Recent research suggested that radiation therapy could significantly elevate the likelihood of developing SPNs among patients with initial lung cancer, breast cancer, nasopharyngeal carcinoma, and prostate cancer [35, 36, 37, 38]. Previous studies reported that external beam radiation therapy, as a common and efficacious treatment for prostate cancer, was linked to a higher risk of developing SPNs of the bladder, lung, and rectum [39, 40, 41]. In addition to their adverse effects, cancer therapies could increase life expectancy and survival, resulting in a higher chance of developing new primary neoplasms among cancer survivors [12]. A study from the US reported that the cumulative incidence of SPNs increased from 7.2% at 5 years to 17.2% after 20‐year survival [42].

The observed significant risk of SPNs in the present study may be explained by the above‐mentioned factors. According to the previous GPCR reports, cancers of the breast, stomach, colorectum, prostate, lung, and esophagus were the most common malignancies in the Golestan population [5, 7, 16]. Different reports from Golestan province suggested a very high incidence of upper gastrointestinal cancers [6, 43, 44] and increasing trends in incidence rates of different types of cancers in this high‐risk area, mainly due to an increase in exposure to risk factors during recent decades [5, 7, 16, 44, 45, 46]. The results of the Golestan Cohort Study suggested that Golestan residents were exposed to cancer‐related risk factors including drinking hot tea, indoor air pollution, drinking unpiped water, opium consumption, smoking, obesity, and agricultural pesticides, which may be related to different types of cancers [47, 48]. Additionally, similar to other studies, cancer survival in the Golestan province has been on the rise in recent years, primarily due to improvements in healthcare access and therapeutic services [11, 49]. Further investigations are recommended to clarify the factors related to the high risk of SPNs among Golestan cancer patients.

Regarding the high risk of developing new neoplasms in cancer patients, designing and implementing effective and efficient surveillance programs for cancer survivors should be considered a priority in healthcare systems [50, 51, 52], especially in high‐risk populations including Golestan province of Iran. As Golestan province is one of the 31 provinces of Iran that makes up about 2% of Iran's total population, and regarding diverse lifestyle and genetic characteristics across different provinces, similar studies are recommended to investigate the epidemiological features of SPNs in other parts of Iran.

Our findings showed a higher risk of SPNs among rural residents compared to urban populations, which is likely attributable to the interplay of socioeconomic disparities, lifestyle factors, environmental exposures, and healthcare access issues. Limited access to healthcare resources and financial constraints in rural areas may contribute to delayed diagnosis and treatment of primary cancers, increasing the risk of SPN development [53]. Additionally, rural populations may be more exposed to specific risk factors (e.g., agricultural risk factors, etc.), which are shared risk factors for different types of cancers, resulting in an increase in the risk of SPNs [54, 55]. Further research is needed to better understand the underlying factors and develop targeted interventions to address this health disparity.

Our findings showed that patients with ovary (SIR = 6.83) and prostate (SIR = 6.72) cancer have a greater chance of developing second primary cancer. Ovary cancer is ranked the eighth most prevalent cancer among women worldwide. Although the incidence rates have been declining in certain regions, such as northern Europe and North America, some studies have shown that individuals diagnosed with ovarian cancer may be at elevated risk for the development of SPNs [56, 57, 58]. A retrospective analysis of SEER data revealed a heightened incidence of SPNs among gynecological cancer survivors, underscoring the necessity for life‐long surveillance [59]. Although current literature lacks specific data on the risk of SPNs in ovarian cancer patients, several factors may contribute to the increased risk of SPNs in this population, including genetic predisposition, common environmental risk factors, and treatment‐related effects [60, 61]. Recent advancements in ovarian cancer treatment have improved patients' survival rates [62, 63], resulting in an increased risk of SPNs in ovarian cancer patients [64].

Developing a second primary cancer in prostate cancer patients can be accounted for by prostate cancer treatment effects, particularly radiotherapy, which increases the risk of a second tumor among prostate cancer survivors. Despite advancements in radiotherapy techniques, the adverse effects of prostate cancer therapies, including second primary cancer among long‐term survivors, remain a concern [65]. Additionally, genetic mutations and family history have been identified as risk factors for SPNs in prostate cancer cases [66, 67]. Prostate cancer is the second most common cancer in men globally [68]. In the Golestan province, Iran, prostate cancer incidence rates have risen from 8.4 per 100,000 in 2004 to 17.6 in 2016, with projections of reaching 25.0 in 2025 [7]. Given the high incidence and increasing trends of prostate cancer and the high risk of developing SPN in prostate cancer patients, appropriate surveillance programs should be developed for prostate cancer survivors in Golestan province.

Our findings demonstrated that SPNs of the ovary and NHL were the most important second neoplasms among our cancer survivors. The risk of ovary neoplasm is more than 8‐fold higher among cancer survivors than in the general population. Developing ovary cancer as the second primary malignancy among cancer patients can be related to a shared genetic mutation. In other words, some cancer patients, for example, female breast cancer cases after 10 years of breast cancer diagnosis, may carry a specific germline mutation that is consistent with ovary cancer [12, 15]. Moreover, the common risk factors between breast and ovary cancers, including hormones and dietary factors, can affect the occurrence of the SPN of ovary among cancer patients [14].

We also found a significantly higher risk of NHL among cancer survivors when compared to the general population. Large tumors and their treatment can weaken the immune system, making it more susceptible to new cancers [69]. This increased risk is particularly evident for NHL, especially after therapies targeting the bone marrow, a common side effect in lymphohematopoietic malignancies [70].

The main study limitation includes short‐term follow‐up, with a median follow‐up time of 3.4 years, and consequently a small number of SPN across sub‐populations. The GPCR was a relatively newly established cancer registry (started in 2004), and this was the main reason for short‐term follow‐up in the present study. By continuing the GPCR activity in the next years, more comprehensive findings with long‐term follow‐up data and a larger number of SPNs may be presented in future GPCR reports.

In conclusion, our findings suggest that cancer patients are at higher risk of getting a new primary cancer when compared to the general population. Designing and implementing comprehensive surveillance programs for cancer survivors should be considered in health policy‐making, especially in high‐risk populations including the Golestan province of Iran. Cancer patients with a higher risk of SPNs, including those with ovary and prostate cancers, should be considered a top priority. Further investigations are warranted to clarify the epidemiological aspects and risk factors of SPNs, as well as to design the most effective surveillance programs for cancer survivors.

## Author Contributions

**Susan Hasanpour‐Heidari:** data curation (equal), formal analysis (equal), writing – original draft (equal). **Shahryar Semnani:** investigation (equal), project administration (equal), writing – review and editing (equal). **Abdolreza Fazel:** investigation (equal), project administration (equal), writing – review and editing (equal). **Mohammad Naeimi‐Tabiei:** investigation (equal), methodology (equal), writing – review and editing (equal). **Mahshid Mehrjerdian:** investigation (equal), methodology (equal), writing – review and editing (equal). **SeyedMehdi Sedaghat:** methodology (equal), validation (equal), writing – review and editing (equal). **Hamideh Sadeghzadeh:** methodology (equal), validation (equal), writing – review and editing (equal). **Faezeh Salamat:** data curation (equal), investigation (equal), writing – review and editing (equal). **Nastaran Jafari‐Delouei:** data curation (equal), investigation (equal), writing – review and editing (equal). **Fatemeh Ghasemi‐Kebria:** data curation (equal), investigation (equal), writing – review and editing (equal). **Honeyehsadat Mirkarimi:** data curation (equal), investigation (equal), writing – review and editing (equal). **Nesa Shokouhifar:** data curation (equal), investigation (equal), writing – review and editing (equal). **Behnoush Abedi‐Ardekani:** methodology (equal), validation (equal), writing – review and editing (equal). **Elisabete Weiderpass:** supervision (equal), validation (equal), writing – review and editing (equal). **Gholamreza Roshandel:** conceptualization (equal), formal analysis (equal), writing – original draft (equal). **Reza Malekzadeh:** conceptualization (equal), supervision (equal), writing – review and editing (equal).

## Conflicts of Interest

The authors declare no conflicts of interest.

## Supporting information


Data S1.

## Data Availability

The data that support the findings of this study are available from the corresponding author (GR) upon reasonable request.
