# Supplementary material for: Risk of Second Primary Neoplasms Among Cancer Survivors: A Population‐Based, Cohort Study in Golestan Province, Northern Iran, 2004–2019
Source: Cancer Med. 2025 May 20;14(10):e70926. doi: 10.1002/cam4.70926 (PMC12090201; doi:10.1002/cam4.70926)
Supplement: Supplementary file 1 — Data S1. [file CAM4-14-e70926-s001.docx]

| **Supplementary Table 1. Numbers (observed and expected) and risks (SIR and AER) of second primary neoplasms (SPNs) in a cohort of adolescent and young adult (AYA) cancer patients (excluding non-melanoma skin cancer) in Golestan, Iran, 2004-2019, by sites of first and second cancer and gender** | | | | | | | | | |
| --- | --- | --- | --- | --- | --- | --- | --- | --- | --- |
| **Variables** | | **Male** | | | | **Female** | | | |
|  |  | **PY** | **Observed / Expected** | **SIR (95% CI)** | **AER (95% CI)** | **PY** | **Observed / Expected** | **SIR (95% CI)** | **AER (95% CI)** |
| **Risk of any SPNs by site of first cancer** | |  |  |  |  |  |  |  |  |
|  | **Esophagus** | 251.2 | 1 / 0.1 | -* | -* | 231.5 | 1 / 0.1 | -* | -* |
|  | **Stomach** | 377.4 | 2 / 0.1 | -* | -* | 302.6 | 2 / 0.1 | -* | -* |
|  | **Colorectum** | 1182.8 | 2 / 0.4 | -* | -* | 983.0 | 9 / 0.5 | **18.00** (6.24 - 29.76) | **86.47** (25.01 - 147.92) |
|  | **Liver** | 98.7 | 1 / 0.0 | -* | -* | 0.0 | 0 / 0.0 | -* | -* |
|  | **Pancreas** | 27.7 | 1 / 0.0 | -* | -* | 0.0 | 0 / 0.0 | -* | -* |
|  | **Larynx** | 106.0 | 1 / 0.0 | -* | -* | 0.0 | 0 / 0.0 | -* | -* |
|  | **Lung** | 469.8 | 4 / 0.1 | -* | -* | 339.1 | 1 / 0.2 | -* | -* |
|  | **Leukemia** | 1038.5 | 3 / 0.3 | -* | -* | 930.2 | 3 / 0.5 | -* | -* |
|  | **Breast** | 93.2 | 1 / 0.0 | -* | -* | 6027.9 | 19 / 2.9 | **6.55** (3.61 - 9.50) | **26.71** (11.49 - 41.93) |
|  | **Ovary** | - | - | - | - | 994.3 | 5 / 0.5 | **10.00** (1.23 - 18.77) | 45.26 (-.97 - 91.49) |
|  | **Prostate** | 0.0 | 0 / 0.0 | -* | -* | - | - | - | - |
|  | **Bladder** | 340.5 | 2 / 0.1 | -* | -* | 0.0 | 0 / 0.0 | -* | -* |
|  | **Brain** | 1333.6 | 3 / 0.4 | -* | -* | 822.4 | 3 / 0.4 | -* | -* |
|  | **Thyroid** | 281.6 | 1 / 0.1 | -* | -* | 1672.2 | 2 / 0.8 | -* | -* |
|  | **NHL** | 918.1 | 5 / 0.3 | **16.67** (2.06 - 31.28) | **51.19** (2.04 - 100.34) | 700.0 | 3 / 0.3 | -* | -* |
|  | **Other** | 3630.3 | 20 / 1.1 | **18.18** (10.21 - 26.15) | **52.06** (27.26 - 76.86) | 4239.1 | 25 / 2.1 | **11.90** (7.24 - 16.57) | **54.02** (29.95 - 78.09) |
| **Risk of site-specific SNPs in cancer survivors (i.e. by site of second neoplasm)** | | | | |  |  |  |  |  |
|  | **Esophagus** | 10158.8 | 2 / 0.1 | -* | -* | 17651.7 | 1 / 0.1 | -* | -* |
|  | **Stomach** | 10158.8 | 1 / 0.2 | -* | -* | 17651.7 | 8 / 0.3 | **26.67** (8.19 - 45.15) | **4.36** (1.16 - 7.56) |
|  | **Colorectum** | 10158.8 | 5 / 0.3 | **16.67** (2.06 - 31.28) | **4.63** (0.18 - 9.07) | 17651.7 | 4 / 0.5 | -* | -* |
|  | **Liver** | 10158.8 | 0 / 0.0 | -* | -* | 17651.7 | 4 / 0.1 | -* | -* |
|  | **Pancreas** | 10158.8 | 0 / 0.0 | -* | -* | 17651.7 | 1 / 0.0 | -* | -* |
|  | **Larynx** | 10158.8 | 2 / 0.0 | -* | -* | 17651.7 | 1 / 0.0 | -* | -* |
|  | **Lung** | 10158.8 | 2 / 0.1 | -* | -* | 17651.7 | 1 / 0.2 | -* | -* |
|  | **Leukemia** | 10158.8 | 8 / 0.4 | **20.00** (6.14 - 33.86) | **7.48** (1.89 - 13.07) | 17651.7 | 2 / 0.6 | -* | -* |
|  | **Breast** | 10158.8 | 1 / 0.0 | -* | -* | 17651.7 | 17 / 2.9 | **5.86** (3.08 - 8.65) | **7.99** (3.03 - 12.94) |
|  | **Ovary** | - | - | - | - | 17651.7 | 5 / 0.5 | **10.00** (1.23 - 18.77) | 2.55 (-.05 - 5.15) |
|  | **Prostate** | 10158.8 | 0 / 0.0 | -* | -* | - | - | - | - |
|  | **Bladder** | 10158.8 | 0 / 0.0 | -* | -* | 17651.7 | 2 / 0.1 | -* | -* |
|  | **Brain** | 10158.8 | 3 / 0.4 | -* | -* | 17651.7 | 3 / 0.4 | -* | -* |
|  | **Thyroid** | 10158.8 | 2 / 0.1 | -* | -* | 17651.7 | 3 / 0.8 | -* | -* |
|  | **NHL** | 10158.8 | 7 / 0.3 | -* | -* | 17651.7 | 7 / 0.3 | **23.33** (6.05 - 40.62) | **3.80** (0.80 - 6.80) |
|  | **Other** | 10158.8 | 14 / 1.0 | **14.00** (6.67 - 21.33) | **12.80** (5.32 - 20.27) | 17651.7 | 14 / 1.8 | **7.78** (3.70 - 11.85) | **6.91** (2.50 - 11.33) |
| PY: Person-years at risk; SIR: Standardized Incidence Ratio; AER: Absolute excess risks; NHL: Non-Hodgkin Lymphoma; 95% CI: 95% confidence interval  * SIR and AER were not calculated if the observed number of SNPs were less than 5. | | | | | | | | | |

Significant SIRs and AERs are marked in **bold**.

| **Supplementary Table 2. Numbers (observed and expected) and risks (SIR and AER) of second primary neoplasms (SPNs) in a cohort of adult cancer patients (excluding non-melanoma skin cancer) in Golestan, Iran, 2004-2019, by sites of first and second cancer and gender** | | | | | | | | | |
| --- | --- | --- | --- | --- | --- | --- | --- | --- | --- |
| **Variables** | | **Male** | | | | **Female** | | | |
|  |  | **PY** | **Observed / Expected** | **SIR (95% CI)** | **AER (95% CI)** | **PY** | **Observed / Expected** | **SIR (95% CI)** | **AER (95% CI)** |
| **Risk of any SPNs by site of first cancer** | |  |  |  |  |  |  |  |  |
|  | **Esophagus** | 6441.8 | 28 / 25.2 | 1.11 (0.70 - 1.52) | 4.35 (-17.85 - 26.54) | 5570.5 | 15 / 18.2 | 0.82 (0.41 - 1.24) | -5.74 (-26.02 - 14.53) |
|  | **Stomach** | 8423.2 | 36 / 33.0 | 1.09 (0.73 - 1.45) | 3.56 (-15.77 - 22.89) | 4031.2 | 14 / 13.2 | 1.06 (0.51 - 1.62) | 1.98 (-23.37 - 27.34) |
|  | **Colorectum** | 6293.5 | 36 / 24.6 | 1.46 (0.99 - 1.94) | 18.11 (-6.13 - 42.36) | 5307.0 | 38 / 17.3 | **2.20** (1.50 - 2.89) | **39.00** (11.54 - 66.47) |
|  | **Liver** | 1069.5 | 8 / 4.2 | 1.90 (0.58 - 3.22) | 35.53 (-28.48 - 99.54) | 709.1 | 5 / 2.3 | 2.17 (0.27 - 4.08) | 38.08 (-36.61 - 112.76) |
|  | **Pancreas** | 876.5 | 7 / 3.4 | 2.06 (0.53 - 3.58) | 41.07 (-31.04 - 113.18) | 559.0 | 4 / 1.8 | -* | -* |
|  | **Larynx** | 1937.2 | 15 / 7.6 | 1.97 (0.97 - 2.97) | 38.20 (-9.90 - 86.30) | 430.2 | 2 / 1.4 | -* | -* |
|  | **Lung** | 5400.0 | 27 / 21.1 | 1.28 (0.80 - 1.76) | 10.93 (-14.25 - 36.10) | 2642.7 | 16 / 8.6 | 1.86 (0.95 - 2.77) | 28.00 (-8.78 - 64.79) |
|  | **Leukemia** | 2520.6 | 15 / 9.9 | 1.52 (0.75 - 2.28) | 20.23 (-18.57 - 59.04) | 1856.3 | 9 / 6.1 | 1.48 (0.51 - 2.44) | 15.62 (-25.41 - 56.65) |
|  | **Breast** | 315.6 | 3 / 1.2 | -* | -* | 15497.4 | 66 / 50.6 | 1.30 (0.99 - 1.62) | 9.94 (-3.72 - 23.59) |
|  | **Ovary** | - | - | - | - | 2451.6 | 23 / 8.0 | **2.88** (1.70 - 4.05) | **61.18** (16.67 - 105.70) |
|  | **Prostate** | 5492.4 | 43 / 21.5 | **2.00** (1.40 - 2.60) | **39.15** (10.49 - 67.81) | - | - | - | - |
|  | **Bladder** | 4586.3 | 29 / 18.0 | **1.61** (1.02 - 2.20) | 23.98 (-5.31 - 53.28) | 1217.2 | 10 / 4.0 | 2.50 (.95 - 4.05) | 49.29 (-10.96 - 109.54) |
|  | **Brain** | 1894.6 | 6 / 7.4 | 0.81 (0.16 - 1.46) | -7.39 (-45.26 - 30.48) | 1694.8 | 11 / 5.5 | 2.00 (0.82 - 3.18) | 32.45 (-14.52 - 79.43) |
|  | **Thyroid** | 571.8 | 5 / 2.2 | 2.27 (0.28 - 4.26) | 48.97 (-43.01 - 140.94) | 1435.3 | 4 / 4.7 | -* | -* |
|  | **NHL** | 2398.6 | 17 / 9.4 | 1.81 (0.95 - 2.67) | 31.69 (-10.30 - 73.67) | 1313.1 | 12 / 4.3 | **2.79** (1.21 - 4.37) | 58.64 (-1.62 - 118.91) |
|  | **Other** | 8546.3 | 49 / 33.5 | **1.46** (1.05 - 1.87) | 18.14 (-2.69 - 38.97) | 10713.2 | 90 / 35.0 | **2.57** (2.04 - 3.10) | **51.34** (30.88 - 71.79) |
| **Risk of site-specific SNPs in cancer survivors (i.e. by site of second neoplasm)** | | | | |  |  |  |  |  |
|  | **Esophagus** | 56768.0 | 28 / 25.7 | 1.09 (.69 - 1.49) | 0.41 (-2.12 - 2.94) | 55428.6 | 22 / 19.1 | 1.15 (0.67 - 1.63) | .52 (-1.74 - 2.79) |
|  | **Stomach** | 56768.0 | 47 / 37.4 | 1.26 (0.90 - 1.62) | 1.69 (-1.48 - 4.86) | 55428.6 | 33 / 15.4 | **2.14** (1.41 - 2.87) | **3.18** (.72 - 5.64) |
|  | **Colorectum** | 56768.0 | 32 / 22.7 | 1.41 (0.92 - 1.90) | 1.64 (-0.92 - 4.19) | 55428.6 | 38 / 17.2 | **2.21** (1.51 - 2.91) | **3.75** (1.13 - 6.38) |
|  | **Liver** | 56768.0 | 5 / 6.7 | 0.75 (0.09 - 1.40) | -.30 (-1.48 - .88) | 55428.6 | 4 / 3.3 | -* | -* |
|  | **Pancreas** | 56768.0 | 7 / 5.3 | 1.32 (0.34 - 2.30) | 0.30 (-0.91 - 1.51) | 55428.6 | 4 / 3.0 | -* | -* |
|  | **Larynx** | 56768.0 | 10 / 6.6 | 1.52 (0.58 - 2.45) | 0.60 (-0.81 - 2.01) | 55428.6 | 2 / 1.7 | -* | -* |
|  | **Lung** | 56768.0 | 24 / 24.2 | 0.99 (0.59 - 1.39) | -0.04 (-2.43 - 2.36) | 55428.6 | 10 / 9.6 | 1.04 (0.40 - 1.69) | 0.07 (-1.49 - 1.64) |
|  | **Leukemia** | 56768.0 | 16 / 10.4 | 1.54 (0.78 - 2.29) | 0.99 (-0.79 - 2.76) | 55428.6 | 13 / 7.0 | 1.86 (0.85 - 2.87) | 1.08 (-0.50 - 2.66) |
|  | **Breast** | 56768.0 | 6 / 1.1 | **5.45** (1.09 - 9.82) | 0.86 (-0.06 - 1.78) | 55428.6 | 55 / 44.0 | 1.25 (.92 - 1.58) | 1.98 (-1.53 - 5.50) |
|  | **Ovary** | - | - | - | - | 55428.6 | 25 / 7.9 | **3.16** (1.92 - 4.41) | **3.09** (1.06 - 5.11) |
|  | **Prostate** | 56768.0 | 36 / 20.0 | **1.80** (1.21 - 2.39) | **2.82** (.23 - 5.40) | - | - | - | - |
|  | **Bladder** | 56768.0 | 17 / 14.0 | 1.21 (0.64 - 1.79) | 0.53 (-1.39 - 2.45) | 55428.6 | 14 / 3.4 | **4.12** (1.96 - 6.27) | **1.91** (0.44 - 3.39) |
|  | **Brain** | 56768.0 | 5 / 7.6 | 0.66 (0.08 - 1.23) | -0.46 (-1.68 - 0.77) | 55428.6 | 8 / 5.8 | 1.38 (0.42 - 2.34) | 0.40 (-0.92 - 1.71) |
|  | **Thyroid** | 56768.0 | 1 / 1.9 | -* | -* | 55428.6 | 7 / 4.4 | 1.59 (0.41 - 2.77) | 0.47 (-0.72 - 1.66) |
|  | **NHL** | 56768.0 | 16 / 7.8 | **2.05** (1.05 - 3.06) | 1.44 (-0.24 - 3.13) | 55428.6 | 10 / 4.2 | 2.38 (0.91 - 3.86) | 1.05 (-0.29 - 2.38) |
|  | **Other** | 56768.0 | 74 / 31.0 | **2.39** (1.84 - 2.93) | **7.57** (4.04 - 11.11) | 55428.6 | 74 / 34.9 | **2.12** (1.64 - 2.60) | **7.05** (3.36 - 10.74) |
| PY: Person-years at risk; SIR: Standardized Incidence Ratio; AER: Absolute excess risks; NHL: Non-Hodgkin Lymphoma; 95% CI: 95% confidence interval  * SIR and AER were not calculated if the observed number of SNPs were less than 5. | | | | | | | | | |

Significant SIRs and AERs are marked in **bold**.
